# The Molecular and Cellular Mechanisms Associated with a Microvascular Inflammation in the Pathogenesis of Heart Failure with Preserved Ejection Fraction

**DOI:** 10.32607/actanaturae.10990

**Published:** 2020

**Authors:** A. G. Ovchinnikov, T. I. Arefieva, A. V. Potekhina, A. Yu. Filatova, F. T. Ageev, S. A. Boytsov

**Affiliations:** National Medical Research Center of Cardiology, Moscow, 121552 Russia

**Keywords:** left ventricular hypertrophy, heart failure with preserved ejection fraction, fibrosis, inflammation, macrophage, lymphocyte

## Abstract

Heart failure with preserved ejection fraction (HFpEF) is a severe disease with an often unfavorable outcome. The prevalence of HFpEF continues to increase, while effective treatment options remain elusive. All the medical strategies used to improve the outcome in a heart failure with reduced ejection fraction proved ineffective in HFpEF, which was probably due to the different mechanisms of development of these two types of heart failure and the diversity of the HFpEF phenotypes. According to the current paradigm of HFpEF development, a chronic mild pro-inflammatory state causes a coronary microvascular endothelial inflammation, with further myocardial fibrosis and diastolic dysfunction progression. This inflammatory paradigm of HFpEF has been confirmed with some evidence, and suppressing the inflammation may become a novel strategy for treating and managing HFpEF. This review summarizes current concepts about a microvascular inflammation in hypertrophied myocardium and provides a translational perspective of the anti-inflammatory and immunomodulatory approaches in HFpEF.

## INTRODUCTION

Approximately half of all patients who suffer a heart failure have a normal ejection fraction. The prevalence of heart failures with preserved ejection fraction (HFpEF), in comparison with heart failures with reduced ejection fraction (HFrEF), increases by 1% annually [1]. According to observational studies, the 5-year survival rate for HFpEF is 50% and every second patient re-enters hospital within six months after the previous hospitalization [1].

Although HFpEF is a very serious condition, effective treatment is still lacking. All of the classes of drugs that improve the HFrEF prognosis (renin-angiotensin blockers, beta-blockers, neprilysin inhibitors) have been found to be ineffective in HFpEF, which is probably due to the difference in the mechanisms of development of the two forms of heart failure. The death of cardiomyocytes leads to HFrEF, while declined left ventricular (LV) relaxation and reduced LV compliance, in which myocardial microvascular inflammation plays a key role, are the main pathophysiological changes resulting in HFpEF. To date, this inflammatory concept is the one supported by most experts [2] and some clinical evidence [3].

**Fig. 1:**
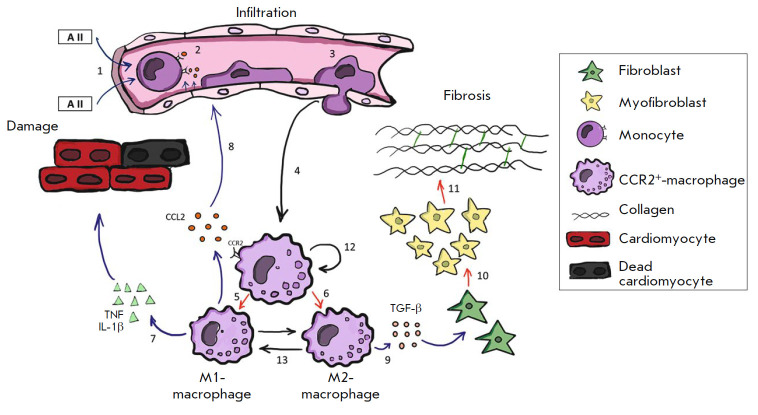
The role of CCR2^+^ macrophages in maintaining a chronic microvascular inflammation in a hypertrophied myocardium. Angiotensin II (A II), which is secreted by a stressed myocardium (1) and CCL2 produced by activated endothelial cells of the coronary microvasculature (2), attract classical monocytes from the bone marrow and spleen into the myocardium. After CCL2-CCR2 interaction with and monocyte invasion into myocardium (3), they convert to CCR2+ macrophages (4). Under the different stimuli, activated CCR2^+^ macrophages further proceed to the M1 (5) or M2 (6) phenotype. M1 macrophages secrete the pro-inflammatory cytokines TNF, IL-1β, and CCL2 that maintain the inflammatory reaction and myocardial damage (7, 8). M2 macrophages are involved in restoring the damaged tissues through the production of pro-fibrotic cytokines (TGF-β) (9). TGF-β stimulates fibroblast-to-myofibroblast differentiation (10) and collagen production, thus promoting fibrosis (11) and diastolic dysfunction progression. The population of CCR2+ macrophages is maintained both due to the migration of monocytes (4) and proliferation *in situ *(12). Under microenvironmental stimuli, macrophages can demonstrate plasticity; e.g., M2-polarized macrophages can be activated and adopt an M1-like phenotype, and vice versa (13). Chronic microvascular myocardial inflammation is characterized by the simultaneous presence of three main stages of the inflammation: infiltration, damage, and repair (fibrosis), which are accompanied by persistent macrophage activation. In an acute inflammation, these three steps usually follow each other

Most HFpEF patients have various concomitant diseases, such as obesity, arterial hypertension, type 2 diabetes mellitus, chronic kidney disease, chronic obstructive pulmonary disease, and anemia [4]. All these diseases, as well as advanced age, are believed to induce and maintain a chronic inflammatory status in the body, which triggers systemic endothelial dysfunction and affects the coronary microvasculature, thus leading to diastolic dysfunction of both ventricles. A myocardial inflammation is a well-studied sequence of discrete immunological events (*Fig. 1*) [5, 6]. Pro-inflammatory cytokines mediate the activation of endothelial cells and thus trigger the whole process. The activated endothelial cells start expressing adhesion molecules on their surface, which interact with the corresponding receptors on circulating monocytes to decelerate the movement of monocytes in the coronary capillaries, to complete stoppage. A high expression of adhesion molecules (intercellular adhesion molecules and E-selectin) is observed in the coronary microvasculature of HFpEF patients, a clear indication of the activation of endothelial cells [7].

Adherence of monocytes to endothelial cells is a necessary condition for the key step in the entire inflammatory process: monocyte migration from the bloodstream into the subendothelial space (*Fig. 1*). This migration is induced by the concentration gradient of chemoattractants, primarily CC chemokine (or monocyte chemotactic protein-1, CCL2/MCP-1) released from the stressed myocardium. Having penetrated the tissue, the monocytes differentiate into macrophages, which start producing the major cytokine in fibrosis, transforming the growth factor β (TGF-β) [7]. TGF-β induces a differentiation of fibroblasts into myofibroblasts; myofibroblasts start producing collagen intensively, which contributes to fibrosis and progressive LV diastolic dysfunction. According to biopsy data, activated macrophages producing high levels of TGF-β accumulate in large amounts in the myocardium of HFpEF patients, which is associated with fibroblast activation and excessive collagen deposition [8, 9].

## MYOCARDIAL INFLAMMATION AND FIBROSIS ARE REGULATED BY MACROPHAGES

In addition to fibrosis, signs of inflammation are always present in experimental models of pressure- overload myocardial hypertrophy [6]. Moreover, the fibrotic and inflammatory areas usually overlap and the more pronounced the inflammation, the more pronounced the fibrosis [6]. Inflammation always occurs earlier than fibrosis, and, if the inflammation is suppressed, then fibrosis is also prevented [6]. Like any other type of inflammation, an inflammation in a hypertrophied myocardium is mediated by innate and adaptive immunity [10, 11], where the key event is monocyte migration from the bloodstream into the subendothelial space, with subsequent differentiation into macrophages. Human monocytes are divided into three subsets based on the expression of CD14 and CD16 proteins: classical monocytes (CD14^++^/ CD16^–^), intermediate monocytes (CD14^++^/CD16^+^), and non-classical monocytes (CD14^+^/CD16^++^). Classical monocytes are the ones that trigger the inflammation in a pressure overload. These monocytes are produced from hematopoietic precursors and stem cells in the bone marrow (there is also a small pool of such cells in the spleen). Cytokines such as the chemokine CCL2/ MCP-1 and granulocyte-macrophage colony-stimulating factor, which are secreted by activated endothelial cells and resident macrophages, induce monocyte recruitment [12, 13]. The classical monocyte count is significantly elevated (two- to fourfold) in HFpEF patients, confirming the systemic pro-inflammatory status [8, 14].

The myocardium contains almost no monocytes; however, a certain number of resident macrophages persist. Macrophages play a key role in regulating matrix protein production and degradation, as well as in inducing fibrosis in many diseases, including HFpEF [15]. Under stress (e.g., in ischemia or pressure overload), the population of macrophages in the myocardium increases significantly due to both the proliferation of resident macrophages and migration of classical monocytes from the bloodstream, with their subsequent differentiation into macrophages (*Fig. 1*) [16, 17].

Resident macrophages and monocyte-derived macrophages (MDMs) differ in function and in their localization in the myocardium. MDMs express CCR2 (the receptor for chemokine CCL2) on their surface (CCR2^+^ macrophages). This chemokine induces a migration of classical monocytes (precursors of CCR2^+^ macrophages) from the bone marrow and spleen to the focus of the inflammation [18]. CCR2^+^ macrophages are predominantly activated through the classical pathway, in the presence of the interferon (INF)-γ produced by type 1 T helper cells and microbial components [19]. CCR2^+^ macrophages play a crucial role in the initiation of an inflammation by producing pro-inflammatory cytokines and acting as antigen-presenting cells for T lymphocytes; therefore, they are considered pro-inflammatory macrophages (or M1 macrophages). CCR2^+^ macrophages are the main cellular component of myocardial infiltrates in a chronic microvascular inflammation (e.g., in HFpEF), when the population of CCR2^+^ macrophages is maintained by both the migration of new monocytes and *in situ *proliferation of cells that have entered the myocardium [12, 16]. CCR2^+^ macrophages contain NLPR3 inflammasomes, which are required for the processing and delivery of interleukin (IL)-1β, the most important inflammatory cytokine, to the stressed myocardium [16]. In contrast to wild-type animals, angiotensin II in CCR2^-^deficient mice was not accompanied by inflammasome activation and interleukin-1β production [20].

Since there is no need for resident macrophages to penetrate into the myocardium, they do not express CCR2 receptors on their surface (CCR2– macrophages). These macrophages renew exclusively through their own proliferation and derive from the embryonic yolk sac [16]. Resident macrophages are responsible for maintaining tissue homeostasis and produce cytokines and growth factors that promote angiogenesis, activation of fibroblasts, collagen synthesis, and inflammation suppression [21]. The so-called M2 macrophages, which differentiate from monocytes with the involvement of IL-4 and IL-13 produced by type 2 T helper cells, have similar properties and transform into reparative macrophages [19]. M1 macrophages are found mainly in fibrotic foci, while M2 macrophages and resident macrophages are usually observed in the viable myocardium adjacent to the microvasculature [12]. The main phenotype of macrophages is believed to depend on their origin. However, it is also possible that the microenvironment affects their functional differentiation. The division of macrophages into a pro-inflammatory and reparative phenotype is rather arbitrary and does not fully reflect their plasticity and the heterogeneity of their properties [21]. Macrophages are sensitive to changes in the microenvironment and can promptly change their functional status from a pro-inflammatory phenotype to a reparative one [22]. Moreover, an entire range of intermediate macrophage subpopulations with different, often heterogeneous, functional activities was revealed using epigenetic and genetic analytical technologies [23].

In 2011, D. Westermann et al. used myocardial biopsy to confirm for the first time that macrophages initiate myocardial fibrosis (MF) in HFpEF patients [9]. Macrophages trigger fibrosis through several mechanisms, such as (1) phagocytosis of dead cells; (2) production of various cytokines, chemokines, and growth factors (primarily TGF-β); and (3) production of tissue metalloproteinase inhibitors that reduce the rate of collagen cleavage [19]. In a stressed myocardium, macrophages synthesize renin and the angiotensin- converting enzyme, thus participating in the local (paracrine) production of angiotensin II, a powerful activator of fibroblasts [24]. In addition to stimulating fibroblasts, angiotensin II also stimulates monocyte release from the bone marrow and spleen [25].

Activated macrophages produce some other stimulators of fibroblast proliferation: galectin-3 and osteopontin. Galectin-3 belongs to the family of soluble beta-galactoside-binding lectins; it can activate myofibroblasts directly [26] and by stimulating the phagocytic activity of macrophages with subsequent TGF-β production [27]. F. Edelmann et al. showed that even a slight increase in the plasma galectin-3 level (on average, from 12.1 to 13.8 ng/mL) in HFpEF patients for 1 year is accompanied by a significantly enhanced MF and worsening of the disease prognosis [28]. Another study in patients hospitalized for heart failure exacerbation demonstrated that the blood level of osteopontin, a fibroblast-activating cytokine, can predict the overall mortality and the rehospitalization risk in HF patients with preserved, but not reduced, EF [29].

Both in ischemic injury and pressure overload, a myocardial inflammation is mediated by MDMs [30]. The intensity of the inflammation in myocardial infarction (MI) is more pronounced than in a pressure overload, which is due to the differences in the severity of inflammatory stimuli. MI is characterized by massive cell death and a rapid, and significant, accumulation of inflammatory cells [31]. In a pressure overload, cardiomyocyte death is minimized, while definite mechanisms of chronic inflammation are under considerations. Possible triggers include cytokines, reactive oxygen species, and the angiotensin II associated with concomitant diseases (obesity, diabetes mellitus, chronic kidney disease, etc.). A hypertrophied myocardium produces a large amount of angiotensin II, which promotes inflammation by stimulating both the formation of reactive oxygen species and the intracellular signaling pathways responsible for NFκB activation [32, 33]. Angiotensin II triggers dendritic cell migration, as well as the proliferation of macrophages and CD4^+^ T lymphocytes via AT1 receptors [34]. In a pressure overload, myocardial cells produce matrix metalloproteinases, which leads to the degradation of collagen fibers and DAMPs (Danger Associated Molecular Patterns) formation. DAMPs interact with the Toll-like receptors of macrophages, activate the NFκB-mediated pathways, and trigger an inflammatory response [35]. The death of cardiomyocytes should also be considered, although it is not as acute and massive as during MI, but sufficient enough to initiate an inflammation in a hypertrophied myocardium [15]. Since all these factors (pro-inflammatory cytokines, angiotensin II, and reactive oxygen species) act continuously, the pressure-overload-induced myocardial inflammation is chronic. All the stages (infiltration, damage, and repair), which usually follow one another in an acute inflammation, are present simultaneously in a pressure-overload-induced myocardial inflammation (fibrosis; *Fig. 1*). Persistent macrophage activation is one of the most characteristic signs of a chronic inflammation.

It remains unclear which microenvironmental factors contribute to the functional rearrangement of macrophages. Such a transformation should necessarily include numerous, and multi-level, intercellular interactions. The key factor in the conversion of macrophages from an inflammatory to a reparative phenotype in MI is the uptake of dead cardiomyocytes by macrophages [36]. When overloaded with cell debris, macrophages decrease their secretion of pro-inflammatory cytokines, such as IL-1β and the tumor necrosis factor (TNF), and start producing pro-fibrotic cytokines, such as IL-10 and TGF-β [36]. Having stimulated the production of collagen and thereby participated in the formation of a post-infarction scar, reparative macrophages undergo apoptosis. In a hypertrophied myocardium, macrophages cannot be turned off and are in constant activation; that is why fibrosis transforms from a compensatory response to an exclusively pathological and poorly controlled process. Healthy human monocytes cultured* in vitro *in a medium containing the serum of HFpEF patients differentiated into macrophages that produced large amounts of the pro-fibrotic cytokine IL-10 [14].

It is worth noting that the same cytokines can be useful in HFrEF but unsafe in HFpEF, a factor that should be considered when developing therapeutic approaches for managing myocardial dysfunction. For instance, in MI, IL-10 is transiently produced by macrophages and actively involved in suppressing the inflammation and repairing damaged tissues, which has the most beneficial effect on post-infarction myocardial healing [37]. On the contrary, stable IL-10 expression potentiates LV dysfunction by stimulating fibrosis under a pressure overload [8]. IL-10 expression in rat myocardium 16 weeks after MI was significantly lower compared to the control group [38]. IL-10 expression was nine times higher in mice with HFpEF developed during normal aging than in younger animals [8]. The pleiotropic effects of IL-10 were likewise observed in another experiment in which the transient expression of IL-10 induced by doxycycline (a tetracycline antibiotic) in mice attenuated the acute lung inflammation caused by bacterial lipopolysaccharide [39]. However, a prolonged (1-month long) overexpression of this cytokine promoted pulmonary fibrosis [40]. Thus, the transition of macrophages from a pro-inflammatory to a reparative phenotype in MI has a protective effect. However, prolonged activation of reparative macrophages in pressure overload ultimately contributes to excessive collagen deposition, increased LV wall thickness, and progression of diastolic dysfunction.

## THE ROLE OF ADAPTIVE IMMUNITY IN THE STRUCTURAL CHANGES IN LEFT VENTRICLE HYPERTROPHY

**Fig. 2:**
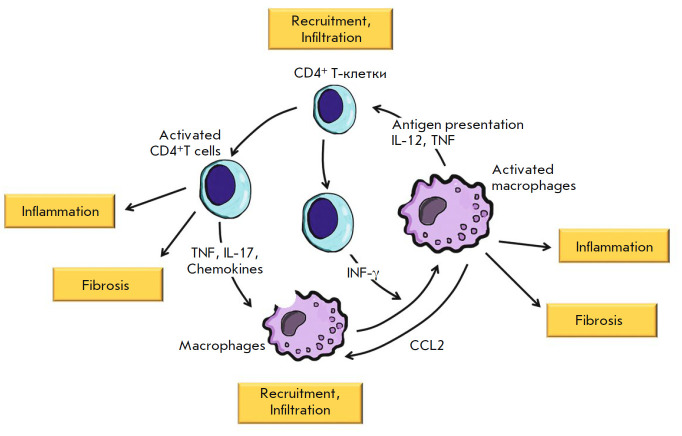
CD4^+^ T lymphocyte and macrophage teamwork in a chronic microvascular inflammation in a hypertrophied myocardium. Activated M1 macrophages secrete TNF and stimulate the mobilization and activation of CD4^+^ T cells. IL-12 produced by M1 macrophages stimulates IF-γ secretion from T cells, further macrophage activation with promoted CCL2 production and monocyte attraction. Activated CD4^+^ T cells produce TNF, IL-17, and chemokines and stimulate macrophage migration. All these cell interactions set up a positive feedback loop, which results in myocardial infiltration with inflammatory cells and chronic inflammation. Activated T lymphocytes and macrophages are involved in microvascular inflammation and myocardial fibrosis

Adaptive immunity plays an important role both in acute myocardial inflammation and post-infarction remodeling [41, 42]. The data on the participation of adaptive immune cells in the structural transformation of the LV in pressure overload is sparse. As already mentioned above, microvascular inflammation in hypertrophied myocardium is chronic. T and B lymphocytes can always be found in the foci of a chronic inflammation, where they actively interact with macrophages and together contribute to the inflammation (*Fig. 2*). Chemokines and cytokines secreted by activated macrophages (mainly TNF and IL-1) induce leukocyte migration to the site of an inflammation. Macrophages act as antigen-presenting cells for T lymphocytes; they express the so-called co-stimulatory molecules on their surface and secrete cytokines (IL-12 and others), which activate T lymphocytes. In turn, the activated T lymphocytes produce cytokines (INF-γ, IL-4, IL-5, and IL-13), which contribute to the activation of macrophages. A vicious circle is established (*Fig. 2*).

T lymphocytes can act as a transmission link between a microvascular inflammation and MF. T. Nevers et al. suggested that T cell migration to the myocardium was an important step in the pathogenesis of hypertensive cardiac remodeling [43]. Transverse aortic constriction (TAC) in T-cell-deficient mice (TCRα-/- line) was not accompanied by CD4^+^ T cell infiltration in the heart. The animals had a normal LV size and contractility, as well as low myocardial levels of intercellular adhesion molecules and brain natriuretic peptide (BNP). In addition, MF was less severe in T cell-deficient than in wild-type mice. Furthermore, wild-type mice with T cell depletion induced byanti-CD3 antibody treatment, immediately after TAC, had a significantly lower severity of LV systolic dysfunction and MF four weeks after surgery.

In another experiment, mice that had undergone TAC showed LV hypertrophy, which is associated with intracardiac activation of CD4^+^ T lymphocytes, while T cell-deficient animals had a much less pronounced myocardial hypertrophy and fibrosis [44]. Genetically determined T and B cell deficiency (RAG2KO mice) was accompanied by significantly less pronounced LV systolic dysfunction, decreased BNP expression in the myocardium, and reduced fibrosis (along with a decreased myocardial infiltration by macrophages). However, all these positive changes completely disappeared after T-cell replenishment [44]. CD4^+^ T cell-deficient mice (MHCIIKO) did not develop LV dysfunction, while mice lacking CD8^+^ T lymphocytes (CD8KO) exhibited the same disease severity as wild-type animals. Mild hypertrophy was also observed in OTII mice (the T lymphocytes in these mice lost their ability to be activated by antigen- presenting cells), which confirms the key role of CD4^+^ T lymphocytes and antigen-presenting cells in MF. Tae Yu et al. in [45] demonstrated the negative effect of CD4^+^ T lymphocytes on cardiac remodeling compared to CD8^+^ T lymphocytes, which is especially important considering the direct cytotoxic effect of CD8^+^ T cells.

The contribution of CD4^+^ T cell activation by antigen- presenting cells in myocardial dysfunction was confirmed by M. Kallikourdis et al. in [46]. The authors were able to prevent T cell stimulation by dendritic cells, B cells, and macrophages using the abatacept immunosuppressant (a selective modulator of the costimulatory signal required for full T cell activation) in the TAC model. This allowed them to maintain normal LV systolic function when prescribing the drug at different time points during TAC and a week after surgery. The positive effect of the drug on the systolic function was accompanied by a decreased BNP expression and reduced MF severity. In addition, a lower content of T cells in the myocardium and a decreased expression of the molecules involved in T cell co-stimulation (e.g., allograft inflammatory factor-1) by activated antigen-presenting cells were also observed.

Regulatory T cells, unlike T helper cells, demonstrate cardioprotective properties and reduce the severity of LV remodeling [47, 48]. Regulatory T cells have an immunosuppressive effect and help maintain immune homeostasis. The lack of regulatory T cells has been shown to result in autoimmune diseases, while a normalized or increased cell number has a positive effect [49]. Regulatory T cells inhibit macrophage activity [50, 51], which allows one to consider them as cells that have the potential to suppress a subacute chronic inflammation in a hypertrophied myocardium and prevent further collagen accumulation. H. Kvakan et al. showed that increasing the population of regulatory T cells by exogenous administration to mice infused with angiotensin II reduces myocardial infiltration by macrophages and prevents the development of fibrosis [52].

Since a chronic inflammation is a prolonged immune response to persistent stimuli, B lymphocytes can play a significant role in hypertensive cardiac remodeling. Activated B cells are usually present in the foci of a chronic inflammation; however, the significance of the antibodies they produce has not been established yet. Most likely, these are autoimmune antibodies to the altered components of the damaged tissue. A. Cordero-Reyes et al. compared mice with severe combined immunodeficiency (SCID) (T cell and B cell-depleted animals) with mice with either B cell or T cell deficiency using infusions of angiotensin II and an endothelial nitric oxide synthase inhibitor [53]. LV remodeling and fibrosis were much more pronounced in wild-type mice (i.e., mice with normal levels of T and B lymphocytes) and mice lacking only T cells than in animals lacking only B lymphocytes and SCID mice. The expression levels of pro-inflammatory cytokines (IL-1β, IL-6, and TNF) were significantly lower in B cell-depleted animals than in B cell-intact mice. Reconstitution of B cells in SCID mice not only enhanced the expression of BNP, IL-1β, IL-6, and TNF, but also promoted LV hypertrophy and fibrosis. The expression of the pro-fibrotic cytokine IL-10 was reduced in all animals with myocardial damage; however, the highest IL-10 levels, which were close to intact myocardium values, were noted in the B cell-deficient group. Areas stained with immunoglobulin (Ig) G3 (a marker of autoimmune myocardial damage) were observed in the myocardial sections of all B cell-intact groups (i.e. wild-type mice, T cell-depleted animals, and SCID mice after B cell reconstitution). In the same study, *in vitro-*activated B lymphocytes stimulated collagen production by myofibroblasts.

Recently, a relationship between B cells and monocyte recruitment has been established. This relationship is likely mediated by the production of CCL7 chemokine by B cells, which promotes the release of monocytes from the bone marrow and their migration to the area of inflammation [54]. Due to their antigen-presenting ability, B cells can modulate the T cell response. This allows one to expect the suppression of T cell activity and prevention of monocyte migration to the myocardium by modification of the B cell behavior. The significance of such B cell-mediated antigen presentation has been shown in several studies [43, 44]. At the same time, T. Guzik et al. revealed the greater importance of T cells in the development of the LV dysfunction associated with pressure overload [55]. The extent of the myocardial damage in T cell-and B cell-deficient mice (RAG-1−/− mice) after angiotensin II infusion was significantly lower than in the control animals. However, reconstitution of T cells (but not B cells) restored full-blown myocardial damage. The role of lymphocytes in the formation and progression of diastolic dysfunction and HFpEF in humans is actively studied. K. Youker et al. demonstrated the involvement of B lymphocytes in the pathogenesis of heart failure using biopsy [56]. In this study, anti-cardiac antibodies and activated complement components were found in the myocardium of most patients with severe heart failure of various etiologies [56].

## ANTI-INFLAMMATORY STRATEGY IN HFPEF

In general, the idea of using immunomodulation to treat heart failures has been actively tested over the past 20 years in numerous clinical trials: COPE-ADHF using corticosteroids, METIS using methotrexate, IMAC using immunoglobulin, and RENEWAL and ATTACH using TNF inhibitors. Unfortunately, the results of these clinical studies which were performed mainly on HFrEF patients proved inconclusive or, at best, contradictory [57]. For instance, the high efficacy of TNF inhibitors demonstrated experimentally [58, 59] was not confirmed in the clinical trials. The trials demonstrated that the competitive inhibitor of the TNF receptors etanercept and achimeric monoclonal antibody against TNF, infliximab, are ineffective in HFrEF and even increase the risk of death in some cases [60, 61]. The failure of these trials was attributed to the excessive TNF antagonism and inhibition of its protective effect in the form of prevented cardiomyocyte apoptosis in stress. Broad-spectrum drugs are considered undesirable in the treatment of heart failure, because more specific effects are required. For instance, targeted suppression of the activity of one of the IL-1 isoforms (β isoforms) with the monoclonal antibody canakinumab in post-infarction patients significantly improved outcomes in the recently completed CANTOS trial [62]. The main reason behind the failure of all the above-mentioned studies is usually considered to be the fact that, in HFrEF, inflammation in the myocardium is detected only at advanced stages of the disease and initiated by reactive changes in response to severe LV systolic dysfunction; while, in earlier stages, remodeling is regulated through the death of cardiomyocytes [63]. In HFpEF, left ventricular remodeling (progression of fibrosis and diastolic dysfunction) originates from chronic microvascular myocardial inflammation. Since any inflammation is mediated by immune cells, the possibility of suppressing and modulating the immune response in HFpEF is being aggressively studied around the world.

One of the earliest inflammatory events that take place in a hypertrophied myocardium is increased CCL2/MCP-1 chemokine production by endothelial cells and resident macrophages. In fact, the entire inflammatory cascade begins with this response. Since, according to the well-known biomedical law, the most effective interventions are those that affect the earliest stages of the pathological (in this case, inflammatory) process, inhibiting CCL2/MCP-1 seems an extremely attractive therapeutic target. In several experimental models of pressure overload, inhibition of its activity through gene manipulation [64] or immunologically (using neutralizing antibodies) [65] prevented MF and improved LV diastolic function.

Inhibiting the CCL2/CCR2 axis in an inflammation not only prevents the migration of CCR2+ monocytes to the myocardium, but can also alter the functional activity of fibroblasts. Angiotensin II infusion in CCR2- or CCL2/MCP-1-deficeint mice not only significantly reduced myocardial infiltration by macrophages, but also decreased the expression of smooth muscle α-actin (a marker of myofibroblast activation), as well as the severity of LV fibrosis and diastolic dysfunction compared to wild-type animals [64, 66, 67]. Interestingly, suppression of inflammation did not affect myocardial hypertrophy in these experiments, which indicates a fundamental difference in the growth stimuli for cardiomyocyte and interstitial compartments: it is a hemodynamic load in the first case and an inflammation in the latter one.

Altering the macrophage phenotype may soon become a major strategy for reducing non-infectious myocardial dysfunction. A fundamentally new way of macrophage polarization, namely intracoronary injection of cardiosphere-derived cells (CDCs), is now being actively studied. CDCs are a specially treated heterogeneous population of stem cells isolated from the myocardium during biopsy. These cells can differentiate into different lineages with anti-inflammatory and anti-fibrotic activity [68]. The protective effect of CDCs is exerted with the involvement of macrophages, as proved by the experiment by de Couto et al., in which clodronate-induced macrophage depletion in rats weakened the ability of CDCs to reduce the MIarea [69]. Administration of CDCs to Dahl salt-sensitive rats fed a high-salt diet reduced the severity of the systemic inflammation and myocardial infiltration by macrophages, which was accompanied by a decreased MF, lower LV filling pressure, reduced pulmonary congestion, and improved overall survival [70]. CDC administration did not affect LV hypertrophy and arterial blood pressure, which once again confirms the leading role played by inflammation and fibrosis in the development of HFpEF. CDCs are believed to produce exosomes (microvesicles) containing “beneficial” microRNAs, which modify the transcriptome of recipient cells [71, 72]. A phase II clinical trial of intracoronary administration of allogeneic CDCs to HFpEF patients is currently underway in the U.S. (clinicaltrials.gov: NCT0294170).

Considering the exceptional phenotypic plasticity of macrophages, approaches aimed at fine-tuning the modulation of macrophages by suppressing their inflammatory and pro-fibrotic activities, but not affecting their ability to maintain myocardial homeostasis and protect against infection, will be most relevant in the future. Alternative strategies, such as the use of nanoparticles delivering a therapeutic load directly to the damaged myocardium, aimed at inhibiting monocyte migration are being actively tested [73, 74]. Small interfering RNAs can act as a therapeutic load [75]. These RNAs can be relatively easily delivered to the phagocytes of the immune system (primarily macrophages) by using nanocarriers, inside which they reach the decision nodes for macrophage polarization and change the transcription of the required genes, thus avoiding the undesirable side reactions typical of broad-spectrum immunomodulation [75].

It was noticed that the treatment of patients for rheumatoid arthritis using anakinra, an interleukin- 1 receptor antagonist, improves heart function. This served as a reason for testing this drug on HFpEF patients. In 2014, the D-HART study was performed in the U.S. to evaluate anakinra effectiveness in patients with HFpEF and a pro-inflammatory status (with a C-reactive protein level > 2 g mg/ dL). Administration of anakinra to 12 patients for 2 weeks was accompanied by a reduced systemic inflammation (a 74% decrease in the level of C-reactive protein) and a statistically significant increase in peak oxygen consumption (by 1.2 mL/kg/min) [76]. Although a longer (12-week) administration of anakinra did not result in increased peak oxygen consumption in the next D-HART-2 trial in 31 patients, it was still associated with a decreased level of blood BNP [77]. To date, it remains unclear how canakinumab, which inhibits the IL-1β isoform and is highly effective in post-infarction patients [62], can improve the diastolic function in HFpEF patients.

The use of such potentanti-inflammatory drugs as anakinra and canakinumab is unsafe because of the risk of side effects, especially in elderly and debilitated patients, who constitute most of the HFpEF patients. Using HMG-CoA reductase inhibitors or statins is much safer in such cases. Although their anti-inflammatory effect is not as strong as that of IL-1 inhibitors, it is sufficient enough to suppress the chronic mild inflammation in the myocardium. It is the case when both the balance between the pathological substrate (chronic mild inflammation) and the strength of the drug effect on this substrate (mild anti-inflammatory effect of statins) can be observed, which is typical of the most effective therapeutic interventions. There is some evidence indicating that statins are effective in HFpEF. According to biopsy results, HFpEF patients taking statins exhibited a lower level of nitrotyrosine (a marker of oxidative processes) in the myocardium, a higher protein kinase G activity, smaller cardiomyocyte size, and lower cardiomyocyte resting tension compared to statin-naive HFpEF patients [63]. In a Russian retrospective cohort study conducted on 223 patients with a compensated (asymptomatic) hypertensive heart disease, the absence of statin therapy was an independent predictor of the subsequent development of HFpEF. On the contrary, administration of statins was associated with a threefold reduction in the risk of HFpEF and a twofold decrease in the risk of progression of LV diastolic dysfunction (increase in its degree) [78]. According to the preliminary data of a Russian prospective single-site clinical study, administration of rosuvastatin and atorvastatin in statin-naive HFpEF patients significantly improves load tolerance, which was accompanied by restoration of the diastolic reserve and a decrease in the LV filling pressure both at rest and during exercise [79]. A recent large meta-analysis showed that administration of statin to patients with a heart failure and > 40% ejection fraction is associated with a significant decrease in total mortality by 15%, cardiovascular mortality by 17% and hospitalization rate due to exacerbation of heart failure by 24% [80]. All the “diastolic” effects of statins (anti-inflammatory effect, anti-fibrotic action, and the effect that improves the endothelial function) depend on the degree of HMG-CoA reductase inhibition in myocardial cells: cardiomyocytes, endotheliocytes, fibroblasts, macrophages, and lymphocytes [81]. The pharmacokinetic properties of statins can also play an important role, among which fat-soluble statins are likely to hold an advantage because of their ability to freely cross the plasma membrane and penetrate various types of cells [82].

## CONCLUSION

HFpEF is a complex pathological condition with various phenotypic manifestations that are caused by a chronic systemic inflammation. The inflammation leads to myocardial fibrosis – the main cause of diastolic dysfunction progression. To prevent or suppress the development of fibrosis, one should first combat the microvascular inflammation. The chronic inflammation in a hypertrophied myocardium is an immunological process that involves innate and adaptive immunity and is associated with the persistent activation of macrophages and myofibroblasts.

The discovery of the key role played by monocytes and macrophages in the progression of hypertrophied myocardium fibrosis made these cells an attractive therapeutic target. To date, experimental studies with a pressure overload have demonstrated the positive character of the outcome of interventions aimed at inhibiting monocyte migration and neutralizing the pro-inflammatory and pro-fibrotic effects of macrophages. Further development of the anti-inflammatory strategy for HFpEF should focus on selective action on macrophages and other immune cells, which will allow one to decrease the left ventricular dysfunction progression without increasing the risk of the side effects associated with immunomodulating approaches.

## Abbreviations

A II: angiotensin II
CDC: cardiosphere-derived cell
HFpEF: heart failure with preserved ejection fraction
IL: interleukin; INF – interferon
LV: left ventricle
MCP-1: monocyte chemoattractant protein-1
MF: myocardial fibrosis
MDM: monocyte-derived macrophage
MI: myocardial infarction
SCID: severe combined immunodeficiency
TAC: transverse aortic constriction
TGF-β: transforming growth factorβ
TNF: tumor necrosis factor
